# Comments on *‘An airway organoid-based screen identifies a role for the HIF1α‒glycolysis axis in SARS-CoV-2 infection’*

**DOI:** 10.1093/jmcb/mjab075

**Published:** 2021-11-30

**Authors:** Xiaohua Duan, Hui Wang, David D Ho, Robert E Schwartz, Todd Evans, Shuibing Chen

**Affiliations:** Department of Surgery, Weill Cornell Medicine, 1300 York Ave., New York, NY 10065, USA; State Key Laboratory of Oncogenes and Related Genes, Center for Single-Cell Omics, School of Public Health, Shanghai Jiao Tong University School of Medicine, Shanghai 200025, China; State Key Laboratory of Oncogenes and Related Genes, Center for Single-Cell Omics, School of Public Health, Shanghai Jiao Tong University School of Medicine, Shanghai 200025, China; Aaron Diamond AIDS Research Center, Columbia University Vagelos College of Physicians and Surgeons, New York, NY 10032, USA; Division of Gastroenterology and Hepatology, Department of Medicine, Weill Cornell Medicine, 1300 York Ave., New York, NY 10065, USA; Department of Physiology, Biophysics and Systems Biology, Weill Cornell Medicine, 1300 York Ave., New York, NY 10065, USA; Department of Surgery, Weill Cornell Medicine, 1300 York Ave., New York, NY 10065, USA; Department of Surgery, Weill Cornell Medicine, 1300 York Ave., New York, NY 10065, USA

Coronavirus disease 2019 (COVID-19) has been an ongoing public health crisis since the end of 2019; besides vaccine development, there have been major research efforts focused on developing antiviral therapeutics. Remdesivir was the first US Food and Drug Administration (FDA)-approved antiviral drug for COVID-19. Subsequently, the FDA granted emergency use authorization (EUA) for three monoclonal antibody treatments, including sotrovimab or a combination of casirivimab and imdevimab, or bamlanivimab and etesevimab, each of which targets the coronavirus spike protein to block viral entry. Most recently, Britain granted conditional authorization for the ribonucleoside analog molnupiravir, developed by Merck as a viral replication inhibitor. The protease inhibitor PF-07321332 developed by Pfizer and boosted by ritonavir showed promising results in a phase III clinical trial, reducing the risk of hospitalization or death by 89% compared with placebo.

Despite tremendous research efforts to combat the pandemic, there remains a need to better understand the viral life cycle and host response in disease-relevant models. Cell-based models have been developed to study viral entry, life cycle, tropism, and pathogenesis. The African green monkey Vero E6 cell line expresses the ACE2 entry receptor and is commonly used to study virus entry and expansion (Hoffmann et al., 2020). The human cell lines HEK293T, Calu-3, Caco-2, and Huh7 are also permissive for viral infection *in vitro* (Chu et al., 2020). However, as these models are derived from human cancers or are quite different from their initial cell of origin, they do not accurately mimic human physiological and pathological responses. Human primary cells (Hou et al., 2020) and adult organoids (Lamers et al., 2020; Salahudeen et al., 2020) may better model SARS-CoV-2 infection, but are limited by the scale for what is required for high-throughput drug screening. Organoids derived from human pluripotent stem cells (hPSCs) overcome such limitations, as they can be used to study infection of relevant normal human tissues and can be scaled for COVID-19 disease modeling and drug discovery (Yang et al., 2020; Duan et al., 2021; Han et al., 2021).

In the recent publication entitled ‘An airway organoid-based screen identifies a role for the HIF1α‒glycolysis axis in SARS-CoV-2 infection’ (Duan et al., 2021), we developed a modified protocol to generate airway organoids from hPSCs (hPSC-AOs). Single-cell RNA sequencing (scRNA-seq) profiling showed that the organoids have a cellular composition similar to the adult human airway counterpart. Morphologically, hPSC-AOs contain beating cilia, a typical characteristic of adult airway tissue. Immunostaining and scRNA-seq data showed that ACE2 is expressed in acetyl-α-tubulin^+^FOXJ1^+^ ciliated-like cells. The hPSC-AOs are permissive to SARS-CoV-2 infection, and the ciliated cells are the main target, consistent with primary tissue data (Hou et al., 2020).

We then performed a high-content chemical screen using hPSC-AOs to identify antiviral drugs. One compound, GW6471, was validated to decrease viral infection in a dose-dependent manner, independent of cytotoxicity. GW6471 also inhibited infection of hPSC-AOs by the B.1.351 SARS-CoV-2 variant, as well as inhibiting infection of hPSC-derived colon organoids. There-fore, GW6471 displays broad-spectrum anti-SARS-CoV-2 activity in multiple tissues.

Transcriptomic and metabolic profiling was applied to investigate the GW6471 mechanism of action. GW6471 was found to inhibit the hypoxia inducible factor 1 subunit alpha (HIF1α) pathway when hPSC-AOs or hPSC-COs were treated after infection. A chemical inhibitor of HIF1α and short hairpin RNAs (shRNAs) targeting HIF1α both validated the essential role of HIF1α in permissiveness of SARS-CoV-2 infection. HIF1α is known as a classic upstream regulator of anaerobic glycolysis. Indeed, metabolic profiling identified decreased glycolysis following GW6471 treatment, consistent also with our transcriptomic profiling. Consistent with our results, a previous study of SARS-CoV-2 infected monocytes suggested that enhanced glycolysis induced by increased HIF1α levels can sustain viral replication (Codo et al., 2020). The higher rate of glycolysis leads to decreased pyruvate metabolism in the mitochondria, which is the rate-limiting intermediate metabolite in the conversion of carbohydrates into fatty acids and cholesterol. Our metabolic pro-filing data also show a lower level of fatty acid synthesis in the GW6471 treatment group. Finally, three compounds tar-geting key steps of fatty acid synthesis, including xanthohumol (an inhibitor of diacylglycerol acetyltransferase) and 5-(tetradecyloxy)-2-furoic acid and ND-646 (two inhibitors of acetyl-coA carboxylase), were confirmed to block SARS-CoV-2 infection. A recent study supports our conclusion that blocking fatty acid synthesis reduces SARS-CoV-2 infection (Chu et al., 2021). Together, transcriptomic and metabolic profiling revealed a key role for the HIF1α‒glycolysis‒fatty acid synthesis axis in mediating productive SARS-CoV-2 infection.

**Figure 1:**
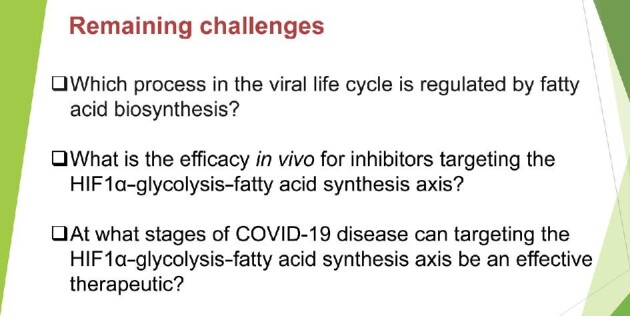
Remaining challenges for the development of therapeutics to inhibit SARS-CoV-2 infection through targeting the HIF1α‒glycolysis‒fatty acid synthesis axis.

In summary, our study presents an hPSC-AO-based model to study the interactions between host human tissues and SARS-CoV-2. By performing a high-content chemical screen and subsequent mechanistic studies, we identified a critical role for the HIF1α‒glycolysis‒fatty acid synthesis axis during SARS-CoV-2 infection, which contains several druggable targets for anti-SARS-CoV-2 drug development. Further studies are needed to develop targeting of the host metabolic state into a clinical therapeutic strategy (Figure 1).

[*R.E.S. is on the scientific advisory board of Miromatrix. T.E. and S.C. are the co-founders of OncoBeat*.]
